# Persistence of Depression and Anxiety despite Short-Term Disease Activity Improvement in Patients with Systemic Lupus Erythematosus: A Single-Centre, Prospective Study

**DOI:** 10.3390/jcm11154316

**Published:** 2022-07-25

**Authors:** Myrto Nikoloudaki, Argyro Repa, Sofia Pitsigavdaki, Ainour Molla Ismail Sali, Prodromos Sidiropoulos, Christos Lionis, George Bertsias

**Affiliations:** 1 Department of Rheumatology and Clinical Immunology, University Hospital of Heraklion and Medical School, University of Crete, 71110 Heraklion, Greece; myrtw.95@hotmail.gr (M.N.); arrepa2002@yahoo.gr (A.R.); sofiamed@windowslive.com (S.P.); aynursalih@msn.com (A.M.I.S.); sidiropp@uoc.gr (P.S.); 2 Institute of Molecular Biology and Biotechnology—FORTH, 71110 Heraklion, Greece; 3 Clinic of Social and Family Medicine, University of Crete Medical School, 71110 Heraklion, Greece; lionis@galinos.med.uoc.gr

**Keywords:** comorbidities, mood disorders, low-disease activity, compliance, patient outcome

## Abstract

Mental disorders such as anxiety and depression are prevalent in systemic lupus erythematosus (SLE) patients, yet their association with the underlying disease activity remains uncertain and has been mostly evaluated at a cross-sectional level. To examine longitudinal trends in anxiety, depression, and lupus activity, a prospective observational study was performed on 40 adult SLE outpatients with active disease (SLE Disease Activity Index [SLEDAI]-2K ≥ 3 [excluding serology]) who received standard-of-care. Anxiety and depression were determined at baseline and 6 months by the Hospital Anxiety and Depression Scale. Treatment adherence was assessed with a self-reported patient survey. Increased anxiety (median [interquartile range] HADS-A: 11.0 [7.8]) and depression (HADS-D: 8.0 [4.8]) were found at inclusion, which remained stable and non-improving during follow-up (difference: 0.0 [4.8] and −0.5 [4.0], respectively) despite reduced SLEDAI-2K by 2.0 (4.0) (*p* < 0.001). Among possible baseline predictors, paid employment—but not disease activity—correlated with reduced HADS-A and HADS-D with corresponding standardized beta-coefficients of −0.35 (*p* = 0.017) and −0.27 (*p* = 0.093). Higher anxiety and depression correlated with lower treatment adherence (*p* = 0.041 and *p* = 0.088, respectively). These results indicate a high-mental disease burden in active SLE that persists despite disease control and emphasize the need to consider socioeconomic factors as part of comprehensive patient assessment.

## 1. Introduction

Patients with Systemic Lupus Erythematosus (SLE) tend to suffer from a variety of physical and mental comorbidities [1,2]. The latter comprise predominantly depression and anxiety disorders with point prevalence rates of 35.0% (95% confidence interval [CI] 29.9–40.3%) and 25.8% (95% CI 19.2–32.9%), respectively [3], although estimations vary according to the metrics and definitions used [3,4]. In the University of California San Francisco Lupus Outcomes Study, depression (defined by the Center for Epidemiologic Studies depression scale) incidence rate was 8.8 per 100 person-years [5] and in another multi-ethnic and racial cohort from the same region, 16% of SLE patients developed depression (based on the Patient Health Questionnaire-8) over an average observation period of 26 months [6]. Anxiety disorder has been less extensively evaluated, nevertheless a small case-control study found increased prevalence in SLE patients when compared to counterparts with rheumatoid arthritis and healthy individuals [7].

Accruing evidence suggests that mental disorders, especially depression, in patients with SLE are associated with multiple adverse outcomes such as fatigue [8], cognitive difficulties [9,10], subclinical atherosclerosis [11], work [12] or functional [13] disability, and reduced health-related quality of life [14,15]. Indeed, severe forms of these disorders can have detrimental effects on daily-life activities and social roles. In a cross-sectional analysis of 80 SLE patients, Nowicka-Sauer et al. [16] found that anxiety and depression collectively explained 43% of illness perception variance. Accordingly, identifying factors contributing to these comorbidities can advance our understanding of their etiology and also rationalize their possible modification towards the improvement of patient well-being. 

In this regard, controversy exists over the relationship between anxiety and depression and SLE disease activity. Thus, active lupus (quantified for example, with the SLE Disease Activity Index [SLEDAI]), especially from the mucocutaneous and musculoskeletal domains, has been correlated with increased depression and anxiety symptoms in some [14,17,18,19]—but not all [15,20,21,22,23]—studies. Likewise, a connection between inflammatory mediators such as lupus autoantibodies and mood disorders has not been consistently shown (reviewed in [24]). Of potential relevance is the association between depression and lower treatment adherence [25,26,27,28], a known driver for lupus flare and activity. This finding, however, lacks extensive confirmation or may be influenced by other factors such as ethnicity [29,30,31,32]. Therefore, evaluation of the frequency and determinants of mental disorders in different regions and clinical settings is important. Importantly, the majority of aforementioned studies had a cross-sectional design or included patients with no prespecified activity level at entry. 

To this end, we carried out a prospective observational study in active and flaring SLE patients who were treated according to standard-of-care, in order to monitor longitudinal changes in depression and anxiety in relation to disease activity. The main hypothesis we sought to test was whether treatment-induced amelioration of the disease would result in the improvement of the aforementioned mental disorders. Taking advantage of our study context of active SLE, we also examined for the possible relationship between anxiety and depressive symptoms with reduced adherence to treatment.

## 2. Materials and Methods

### 2.1. Study Population

A prospective observational (non-interventional) study was performed at the outpatient clinics of the Department of Rheumatology and Clinical Immunology, University Hospital of Heraklion (Crete, Greece), covering from primary to tertiary care [33,34]. Patients were enrolled by consecutive sampling techniques between May 2021 and September 2021. Inclusion criteria were: (a) SLE diagnosis according to physician assessment and ascertained by the 2019 European Alliance of Associations for Rheumatology (EULAR) and American College of Rheumatology (ACR) classification criteria [35]; (b) age 18–65 years; (c) active disease defined by a clinical (excluding serology) SLEDAI-2K ≥ 3 [36] not present in the previous visit; (d) permanent residence in Crete; and (e) comprehension of Greek language. Patients with other coexisting rheumatic diseases, active neuropsychiatric lupus (diagnosed according to multidisciplinary approach as described elsewhere [37]), dementia, malignancy (past or present), and ongoing pregnancy were excluded. A total of 117 patients visited the outpatient clinics during the enrolment period, 50 of whom met the inclusion criteria. Ten participants did not attend their scheduled follow-up visit, thus data from 40 participants were analyzed.

### 2.2. Monitoring Protocol, Disease Evaluation, and Data Collection

Patients were monitored at two to four-month intervals over a period of six months as part of routine clinical practice and according to disease severity (based on physician judgment). Disease assessment at baseline and during follow-up included: (a) laboratory (complete blood count, liver and renal function, urinalysis) and immunological [serum anti-dsDNA, C3, C4, antiphospholipid antibodies] tests, (b) disease activity (quantified by the SLEDAI-2K [38] and the Safety of Estrogens in Lupus Erythematosus, National Assessment (SELENA)-SLEDAI Physician Global Assessment [PGA] [39]), (c) organ damage (quantified by the Systemic Lupus International Collaborating Clinics (SLICC) and ACR damage index [SDI] [40]), (d) comorbid diseases (ascertained by medical history, chart review and electronic prescription data) and, (e) use of medications, including the route of administration and dosage of glucocorticoids. Data on sociodemographic factors (age, disease duration, education level, marital status) were retrieved from medical charts and verified by patient interviews. Working status was assessed as described elsewhere [41] and included past (never or ever had paid employment) and current working status (having paid employment or not). Data were entered into a secure electronic database installed on the Department of Rheumatology and Clinical Immunology (University Hospital of Heraklion) protected server and network. The operation and maintenance of the database were strictly supervised by the scientifically accountable protocol and access was granted only to authorized users and researchers. All principles of anonymity, confidentiality, and non-traceability of data were adhered to.

### 2.3. Assessment of Anxiety, Depression, and Treatment Adherence

Anxiety and depression levels were determined at baseline and during follow-up by the Hospital Anxiety and Depression Scale (HADS), a self-rating psychometric instrument widely used in SLE [4,24,42] and validated in Greek patients [43] (including patients with chronic rheumatic diseases [44]). Briefly, HADS includes seven questions for each disorder (anxiety, depression), with a score ranging from 0–21. Scores ≤ 7 correspond to normal levels of anxiety or depression, 8–10 to borderline pathological levels, and 11–21 to pathological levels. Patients with a diagnosis of anxiety disorder or depression were identified by reviewing the medical history, formal psychiatric evaluations, use, and indications for anxiolytic or antidepressant treatments (i.e., prescribed for underlying mental disorder as opposed to other conditions such as fibromyalgia). Treatment adherence was estimated with a methodology based on self-reported patient survey (modified from [45]). The scale is calculated by assigning one point for each positive answer, thus ranging from 0 (highest adherence) to 4 (lowest adherence).

### 2.4. Statistical Analysis

Categorical data are presented as numbers with percentages and continuous data as mean with standard deviation (continuous variables) or median with interquartile range (ordinal variables). Linear regression was used to identify factors associated with anxiety and depression. Possible predictors were first assessed by univariate analysis and variables associated with *p*-Value < 0.100 were considered for multivariate-adjusted analysis (stepwise backward selection method). To determine longitudinal changes (follow-up vs. baseline) in disease activity (SLEDAI-2K), anxiety (HADS-A), and depression (HADS-D), we applied the Wilcoxon Signed Rank test. In addition, absolute differences (Δ(*delta*) = follow-up *minus* baseline scores) were calculated for SLEDAI-2K, HADS-A, and HADS-D. Patients were grouped as having stable or worsening, or improving anxiety and depression (ΔHADS-A/D ≥ 0 vs. < 0, respectively) and independent samples Mann–Whitney test was used to examine for between-group differences in ΔSLEDAI-2K. We also used the Spearman correlation test for the correlation of longitudinal changes in anxiety and depression. The association between treatment adherence and baseline anxiety or depression levels was evaluated by a chi-squared test. Statistical significance was indicated as a two-tailed *p*-Value < 0.05. All statistical analyses were performed using SPSS V25.0.

### 2.5. Ethical Aspects

The study was approved by the Research Ethics Committee of the University of Crete and by the Ethics Committee of the University General Hospital of Heraklion, Crete. Written informed consent was obtained from all patients. All conditions for the protection of personal data and medical confidentiality were met.

## 3. Results

### 3.1. Patients with Active SLE Manifest Increased Anxiety and Depression Levels That Persist over Time

We evaluated 40 SLE patients (39 women) with an average (SD) age and disease duration of 50.5 (10.3) and 10.3 (7.0) years, respectively (Table 1 and Supplementary Table S1).

Fourteen patients (35.0%) had high- or tertiary-level education and the majority (52.5%) were engaged in paid employment. A variety of comorbid conditions were present in our study sample, including mental disorders previously diagnosed by a specialist (depression in n = 13 patients). Organ damage (defined as SDI 0) had accrued in 18 (45.0%) patients (Table 1). 

At inclusion, all patients had active disease with a median (IQR) SLEDAI-2K of 6.0 (4.0) (Table 2). Assessment of mental status by the HADS index indicated an increased burden of both anxiety (HADS-A) and depression (HADS-D) with corresponding median (IQR) scores of 11.0 (7.8) and 8.0 (4.8). Accordingly, anxiety and depression of even a mild degree were detected in 70.0% and 52.5% of our patient cohort, respectively. 

According to physician judgment and in line with standard clinical practice, patients were offered with treatment modifications due to active disease including initiation or dosage increase of hydroxychloroquine (n = 1), methotrexate (n = 8), azathioprine (n = 3), mycophenolate (n = 3), cyclophosphamide (n = 1), biological agent (n = 6), and glucocorticoids (n = 14). At the follow-up assessment, a significant reduction was noted in SLEDAI-2K, which reached a median of 4.0 (2.0) (Table 2). Conversely, neither anxiety (HADS-A) nor depression (HADS-D) showed significant trends. Thus, average changes in anxiety and depression scores were minimal (median [IQR]: 0.0 [4.8] and −0.5 [4.0], respectively). These results indicate that despite a short-term lowering of disease activity, the burden of mental disorders tends to remain stable and non-improving in patients with SLE.

### 3.2. Lack of Correlation between Longitudinal Changes in Disease Activity and Mental Disorders in SLE Patients

**Figure 1:**
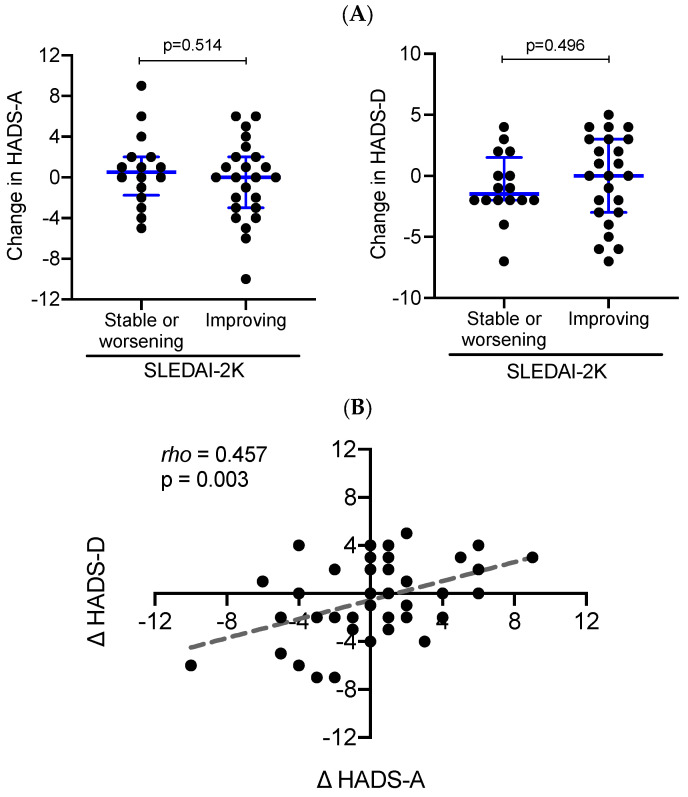
Longitudinal changes in anxiety and depression in association with improvement or not in disease activity. (**A**) Dot plots demonstrate changes (follow-up *minus* baseline) in HADS-A (left panel) and HADS-D (right panel) in SLE patients with improving vs. stable or worsening disease activity (SLEDAI-2K). Independent samples Mann–Whitney test was performed between the two patient groups. Blue lines represent medians (interquartile range). (**B**) Correlation of longitudinal changes (follow-up minus baseline) in HADS-A (Δ HADS-A) and HADS-D (Δ HADS-D) values in the SLE sample (each patient is represented by a separate black circles). The Spearman’s correlation coefficient *rho* = 0.457 (*p*-Value = 0.003).

We sought to gain additional insights into the relationship between anxiety, depression, and disease activity in patients with SLE. Further to examining average trends, we grouped our study sample according to whether SLEDAI-2K was improved (by at least one unit; n = 24) or not (n = 16) during follow-up. We then compared the longitudinal changes in anxiety and depression levels (HADS-A and -D at follow-up minus HADS-A and -D at inclusion visit) between the two aforementioned patient subsets. Comparable changes in HADS-A and HADS-D scores were noted in SLE patients with improved vs. non-improved disease activity (Figure 1A). 

In addition, we identified patients who attained a low-disease activity state according to the definitions proposed by Franklyn et al. [46] and Polachek et al. [36]. Again, HADS-A and HADS-D temporal trends did not differ significantly in patients who achieved or did not achieve low-disease activity (Supplementary Table S2). To address any confounding effects of administered treatments, the previous analyses were repeated separately in patients who were started on or received an increased dose of glucocorticoids due to active disease. In an ancillary analysis, we classified patients according to whether their level of anxiety or depression (as defined in Table 2) improved (for instance, from “severe” to “moderate”), remain stable, or worsened (for instance, from “mild” to “moderate”). By comparing the three aforementioned groups for corresponding changes in SLEDAI-2K, we found no significant trends (Supplementary Table S3).

Notwithstanding the small sample size, results were similar to the whole patient cohort (data not shown). Notably, longitudinal changes in anxiety showed a strong correlation (rho = 0.457, *p* = 0.003) with corresponding changes in depression levels (Figure 1B). Altogether, these data reiterate that SLE patients whose disease improved and even reached a low-activity state, are still burdened with mental disorders.

### 3.3. Association of Mental Disorders with Sociodemographic Characteristics in SLE Patients

The previous findings prompted us to search for other possible predictors of mental disorders in our study sample. To this end, we examined the baseline (i.e., registered at inclusion visit) scores of HADS-A and HADS-D in relationship with standard sociodemographic and clinical parameters. Using previously recommended cut-offs, we found no significant differences in average age, SLE duration, disease activity or severity (SLEDAI-2K), organ damage, presence of comorbidities, and education level in patients with high anxiety (HADS-A ≥ 11) or depression (HADS-D ≥ 8) levels as compared to their counterparts with lower scores (Supplementary Table S4). Conversely, patients with lower levels of anxiety reported paid employment at a significantly higher frequency than those with high anxiety (73.7% vs. 33.3%, respectively, *p* = 0.011). A similar trend in active employment status was observed in SLE patients with low when compared to high-depressive symptoms (68.2% vs. 33.3%, respectively, *p* = 0.028) (Supplementary Table S4). Next, the same parameters were analyzed by linear regression resulting in comparable findings although the association between employment status and HADS-D scores did not reach statistical significance (Table 3). 

Coupled with our aforementioned results, this analysis suggests that socioeconomic factors (employment)—rather than disease activity—may be linked to the excessive burden of mental disorders in patients with SLE.

### 3.4. Increased Anxiety and Depression Levels Are Associated with Lower Adherence to Treatment

Our study focused on trends of anxiety and depression in the context of active lupus. Notably, previous studies have associated mental disorders with poor treatment compliance in patients with SLE [25,26,27,28]. Using a self-reported measure, we found that 19 out of 40 patients (47.5%) had low or very low adherence to treatment. We then investigated whether the severity of mental disorders (assessed at the inclusion visit) correlated with treatment adherence. Within patients with low-anxiety levels (HADS-A < 11), the majority (68.4%) had high compliance; in contrast, among patients with high anxiety (HADS-A ≥ 11), only 38.1% had high compliance and 23.8% exhibited very low or no adherence to treatment (*p* = 0.041; Table 4). 

This relationship was confirmed by a statistically significant positive correlation between HADS-A and adherence scores treated as continuous variables (Spearman’s rho = 0.324, *p* = 0.041) (data not shown). Likewise, SLE patients with lower severity of depressive symptoms (HADS-D < 8) had better treatment compliance (58.1% with high compliance) when compared to those with HADS-D ≥ 8 (33.3%), however, this association was not statistically significant (*p* = 0.088) probably due to the small sample size. Altogether, active SLE patients with a high burden of mental disorders are less likely to adhere to treatment of their disease.

## 4. Discussion

Mental comorbidities such as anxiety and depression are common in patients with SLE, however their association with underlying activity and likewise, their responsiveness to disease improvement remains inconclusive [24]. Our longitudinal analysis of 40 active lupus patients who received standard-of-care treatment to control their disease, demonstrated a high burden of anxiety and depression that remains unchanged at least over a short-term follow-up period and may be determined by socioeconomic factors such as employment status rather than by clinical parameters. Notably, increased levels of anxiety and depression tended to correlate with lower treatment adherence, an established determinant for disease flares [47,48], thus further emphasizing the importance of assessing mental disorders and associated risk factors as part of a comprehensive management plan in patients with SLE. 

In our sample comprising of active SLE patients with an average age and disease duration of 50.5 and 10.3 years, respectively, significant anxiety (HADS-A ≥ 11) and depression (HADS-D ≥ 8) was each noted in 52.5%. This is in line with the results from previous cross-sectional observational studies [7,11,18,23,49,50,51,52] and meta-analyses of published data [3,4], although reported rates may vary according to the study design, population characteristics, and diagnostic instruments used. In the same context, a large Danish cohort study found that compared with the general population, the adjusted hazard ratio of depression was 2.22 (95% CI 1.77–2.77) for SLE patients [53]. Intriguingly, Roberts et al. [54] analyzed data from 194,483 women and found that a history of depression was linked to increased risk (adjusted hazard ratio 2.45; 95% CI 1.74–3.45) for subsequent development of SLE, irrespective of the effect of other confounding factors, thus suggesting a possible cross-interaction between the two conditions. 

Although it is plausible to consider inflammation as a determining factor for mental disorders in SLE [55], there are conflicting reports regarding the relationship of disease activity with anxiety and depression [14,15,17,18,19,20,21,22,23]. To overcome the cross-sectional design limitations of most aforementioned studies, we enrolled active SLE individuals according to predetermined criteria and monitored them at two consecutive time points, i.e., at inclusion and six months post-treatment modification. Contrary to SLEDAI which was significantly improved over time, HADS-A and -D scores remained unchanged. Additionally, we found no reduction in mental disorders within patients who attained a state of low-lupus activity. Subgroup analysis according to intake or not of glucocorticoids yielded similar findings, thus reducing the possibility for a treatment confounding effect [56]. Our results are in agreement with a longitudinal study of 139 SLE patients which revealed four distinct anxiety trajectories that remained stable and not affected by disease activity over an average period of 30.9 months [57]. A similar analysis focusing on depression also showed persistence over time and a lack of association with temporal trends in SLEDAI-2K (average follow-up of 30.2 months) [58]. Collectively, and in line with a previous cohort study indicating that depression might be a long-term outcome of SLE [53], these data suggest that fluctuations of disease activity might not be major drivers of anxiety and depression, especially in the context of long-standing disease, although it has been argued that prolonged remission (i.e., lasting at least 5 years) might have a positive impact on depression [59]. 

Our previous finding coupled with the lack of association between other clinical characteristics and mental disorders prompted us to explore the possible role of sociodemographic factors. We found paid employment status to be protective against both anxiety and depression with corresponding odds ratios of 0.18 and 0.23, independent of SLE severity measures such as SLEDAI and organ damage. This is in agreement with other studies that have identified socioeconomic factors, in particular unemployment, financial strain, or low-social support, as significant correlates of depression in SLE [5,14,19,23,60]. Indeed, mediation modeling has suggested that low-socioeconomic status may impact negatively on the psychosocial resilience [60] and perceived stress [13] of lupus patients, thus contributing to higher anxiety, depression, and subsequent disability. It might be also that some SLE individuals are unable to (find) work due to the severity of the underlying disease or the concomitant anxiety or depressive symptoms [19]. These data underscore the importance of considering relevant socioeconomic factors when assessing the mental status of patients with SLE. 

To our knowledge, our study is the first to evaluate medication adherence in Greek individuals with SLE. Using a self-reported survey, we found that 47.5% of patients with active lupus had low or very low compliance to treatment, a percentage that falls within the range (typically, 43–75%) of previously reported adherence rates [61]. Notably, increased levels of mental disorders tended to correlate with non-adherence, an association that has been previously shown especially for depression in several observational studies [25,26,27,28,62,63]. In this regard, anxiety and depression have been recognized as major determinants of the resilience [29] and illness perception [16] of lupus patients, which can both impact on compliance. Considering the prognostic implications of treatment adherence in terms of flares prevention and improved patient outcomes [64], these findings underline the importance of identifying and managing mental disorders in patients with SLE. 

Several study limitations should be discussed such as that our results were derived from patients with distinct ethnic, demographic, and clinical characteristics, thus may not be generalizable to the whole SLE spectrum. Nevertheless, we applied specific inclusion criteria for active disease evaluated before and after treatment modifications, which facilitates the homogeneity of our data. Although the sample size can be considered relatively small to detect modest effect sizes, our prospective design enabled the generation of robust data regarding intra-individual temporal changes in SLE activity and mental disorders. Because our cohort was followed for six months, we were not able to examine the possible effect of sustained disease control on anxiety and depression. Additionally, the levels of mood disorders prior to study enrolment and how this might have affected the study findings was not available. Finally, the association between mental disorders and employment might be confounded by other parameters not captured in our analysis, still, the validity of our findings has been confirmed by other studies [5,14,19,23,60].

## 5. Conclusions

Active SLE patients exhibit a significant burden of anxiety and depressive symptoms, which remain unchanged despite treatment-induced short-term improvement in disease activity. This concurs with the fact that socioeconomic factors such as employment status, rather than clinical parameters, are significant predictors of the mental status of these patients. Despite the lack of association with disease activity, higher levels of anxiety and depression tend to coincide with lower treatment adherence, which is an established driver of adverse disease outcomes and flares. Together, our findings reiterate the importance of a comprehensive risk assessment for mental disorders in patients with SLE towards the improvement of their overall health status and prognosis.

## Figures and Tables

**Table 1:** Demographic and clinical characteristics of SLE patients (*n =* 40).

	No. (%) or Mean (SD) ^1^
Gender (female)	39 (97.5%)
Race (white)	40 (100.0%)
Age (years)	50.5 (10.3)
Disease duration	10.3 (7.0)
Education level	
Basic or primary	6 (15.0%)
Secondary	19 (47.5%)
High or tertiary	14 (35.0%)
Employment status (working)	21 (52.5%)
Comorbidities	
Hypertension	7 (17.5%)
Dyslipidemia	11 (27.5%)
Osteoporosis	9 (22.5%)
Thyroiditis	7 (17.5%)
Hypothyroidism	5 (12.5%)
COPD ^2^ or bronchial asthma	2 (5.0%)
Diabetes mellitus	2 (5.0%)
Fibromyalgia	15 (37.5%)
Mental disorder	16 (40.0%)
Depression	13 (32.5%)
Anxiety disorder	5 (12.5%)
Organ damage (SDI) ^3^	18 (45.0%)

^1^ SD, standard deviation; ^2^ COPD, chronic obstructive pulmonary disease; ^3^ SDI, SLICC/ACR damage index.

**Table 2:** Disease activity, anxiety, and depression levels in SLE patients at inclusion and follow-up visits.

	Baseline ^1^	Follow-Up	*p*-Value ^2^
**SLEDAI-2K** ^3^	6.0 (4.0)	4.0 (2.0)	0.001
0	0 (0.0%)	5 (12.5%)	
1–4	14 (35.0%)	22 (55.0%)	
5–8	22 (55.0%)	13 (32.5%)	
≥9	4 (10.0%)	9 (0.0%)	
**HADS-Anxiety**	11.0 (7.8)	11.0 (5.5)	0.964
Normal (≤7)	12 (30.0%)	8 (20.0%)	
Mild (8–10)	7 (17.5%)	9 (22.5%)	
Moderate (11–14)	11 (27.5%)	13 (32.5%)	
Severe (≥15)	10 (25.0%)	10 (25.0%)	
**HADS-Depression**	8.0 (4.8)	8.0 (6.8)	0.463
Normal (≤7)	19 (47.5%)	19 (47.5%)	
Mild (8–10)	12 (30.0%)	13 (32.5%)	
Moderate (11–14)	6 (15.0%)	7 (17.5%)	
Severe (≥15)	3 (7.5%)	1 (2.5%)	

^1^ Data are presented as median (interquartile range) or no. (%). ^2^ Wilcoxon Signed Rank Test. ^3^ SLE Disease Activity Index-2K.

**Table 3:** Anxiety and depression in association with sociodemographic and clinical characteristics of SLE patients.

	Anxiety Level (HADS-A)		Depression Level (HADS-D)	
Univariate Analysis	Standardized *β* Coefficient; *p*-Value ^1^			
Age (years)	0.05	0.771	0.04	0.812
Education ^2^	−0.14	0.389	−0.22	0.172
SLE duration (years)	0.10	0.528	0.06	0.721
Employment ^3^	−0.42	0.007	−0.27	0.093
Comorbidities (no.)	0.12	0.466	0.22	0.167
SLEDAI-2K	−0.04	0.786	−0.15	0.353
Organ damage (SDI)	0.08	0.632	−0.22	0.174
SLE treatment				
HCQ ^4,5^	−0.07	0.632	0.11	0.489
Glucocorticoids ^4^	−0.06	0.372	−0.22	0.180
Immunosuppressives ^4^	−0.45	0.003	−0.17	0.284
Biologics ^4^	−0.05	0.765	0.07	0.686
**Multivariable-adjusted** ^5^				
Employment (working) ^3^	−0.35	0.017	−0.27	0.093
Immunosuppressives ^4^	−0.39	0.008	–	–

^1^ Linear regression analysis. 95% CI (95% confidence interval); ^2^ Treated as ordinal variable (0 = primary level; 1 = secondary levels; 3 = tertiary level); ^3^ Treated as dummy variable (1 = paid employment; 0 = not paid employment); ^4^ Treated as dummy variable (1 = use; 0 = no use); ^5^ Backwards elimination model (variables with univariate *p*-Value 0.100 were entered); HCQ, hydroxychloroquine.

**Table 4:** Association of anxiety and depression with treatment adherence in SLE patients.

	Treatment Adherence (Self-Reported): Highest to Lowest			
	**0–1**	**2**	**3–4**	***p*-Value** ^1^
**Anxiety level**				
No or low	13 (68.4%)	6 (31.6%)	0 (0.0%)	
Moderate or severe ^2^	8 (38.1%)	8 (38.1%)	5 (23.8%)	0.041
**Depression level**				
No or low	18 (58.1%)	11 (35.5%)	2 (6.5%)	0.088
Moderate or severe ^3^	3 (33.3%)	3 (33.3%)	3 (33.3%)	

^1^ Chi-squared test. ^2^ HADS-A ≥ 11. ^3^ HADS-D ≥ 8.

## Data Availability

Data are available upon request.

## Supplementary Materials

The following supporting information can be downloaded at: https://www.mdpi.com/2077-0383/11/15/4316/s1, Table S1: Treatment of SLE patients included in the study; Table S2. Longitudinal changes in anxiety and depression in SLE patients who achieved or did not achieve a state of low-disease activity; Table S3: Reclassification of the anxiety and depression level in association with longitudinal change in disease activity in SLE patients; Table S4: Anxiety and depression in association with sociodemographic and clinical characteristics of SLE patients.
