# Correction: Nikoloudaki et al. Persistence of Depression and Anxiety despite Short-Term Disease Activity Improvement in Patients with Systemic Lupus Erythematosus: A Single-Centre, Prospective Study. *J. Clin. Med.* 2022, *11*, 4316

**DOI:** 10.3390/jcm12010227

**Published:** 2022-12-28

**Authors:** Myrto Nikoloudaki, Argyro Repa, Sofia Pitsigavdaki, Ainour Molla Ismail Sali, Prodromos Sidiropoulos, Christos Lionis, George Bertsias

**Affiliations:** 1 Department of Rheumatology and Clinical Immunology, University Hospital of Heraklion and Medical School, University of Crete, 71110 Heraklion, Greece; 2 Institute of Molecular Biology and Biotechnology—FORTH, 71110 Heraklion, Greece; 3 Clinic of Social and Family Medicine, University of Crete Medical School, 71110 Heraklion, Greece

## Text Correction

There was an error in the original publication [1]. In Abstract, “Morisky Medication Adherence Scale-4” has been changed to “a self-reported patient survey”.

A correction has been made to Section 2.3 as follows: “Treatment adherence was estimated with a methodology based on self-reported patient survey (modified from [45])”.

Additionally, in Section 3.4, paragraph 1, a correction has been made as follows: “Using a self-reported measure, we found that 19 out of 40 patients (47.5%) had low or very low adherence to treatment. […] Within patients with low-anxiety levels (HADS-A < 11), the majority (68.4%) had high compliance”. 

In Section 3.4, paragraph 2, “This relationship was confirmed by a statistically significant positive correlation between HADS-A and adherence scores treated as continuous variables (Spearman’s rho = 0.324, *p* = 0.041) (data not shown). Likewise, SLE patients with lower severity of depressive symptoms (HADS-D < 8) had better treatment compliance (58.1% with high compliance) when compared to those with HADS-D ≥ 8 (33.3%)”.

In Section 4, paragraph 5, “the validated MMAS-4 instrument” has been changed to “a self-reported survey”.

Reference correction: remove the ref. 45. With this correction, the order of some references has been adjusted accordingly.

## Error in Table

In the original publication, there was a mistake in Table 4. The header “MMAS-4 Score” has been changed to “Treatment adherence (self-reported): highest to lowest”.

The authors apologize for any inconvenience caused and state that the scientific conclusions are unaffected. This correction was approved by the Academic Editor. The original publication has also been updated.
