# Supplementary material for: Persistence of Depression and Anxiety despite Short-Term Disease Activity Improvement in Patients with Systemic Lupus Erythematosus: A Single-Centre, Prospective Study
Source: J Clin Med. 2022 Jul 25;11(15):4316. doi: 10.3390/jcm11154316 (PMC9329785; doi:10.3390/jcm11154316)
Supplement: Supplementary file 1 [file jcm-11-04316-s001.zip › jcm-1785249-supplementary.pdf]

Supplementary Table S1. Treatment of SLE patients included in the study

	No. patients (%)
SLE treatment	
Glucocorticoids	12 (30.0%)
Hydroxychloroquine	32 (80.0%)
Methotrexate	16 (40.0%)
Cyclosporin A	1 (2.5%)
Azathioprine	6 (15.0%)
Mycophenolate	5 (12.5%)
Leflunomide	2 (5.0%)
Belimumab	9 (22.5%)
Treatment of comorbidities	
Antihypertensives	6 (15.0%)
β-blocker	4 (10.0%)
Lipid lowering agent	6 (15.0%)
Antiplatelet/anticoagulant	3 (7.5%)
Biphosphonate	5 (12.5%)
Antidepressant	13 (32.5%)
Anxiolytic	4 (10.0%)

Supplementary Table S2. Longitudinal changes in anxiety and depression in SLE patients who achieved or not a state of low disease activity

		Baseline [1]	Follow-up [1]	P value [2]
**LLDAS** [3]				
No (n=16)	HADS-A	13 (8)	11 (4)	0.637
	HADS-D	8 (5)	8 (4)	0.916
Yes (n=24)	HADS-A	10 (6)	10 (6)	0.834
	HADS-D	8 (6)	9 (7)	0.303
**LDA** [4]				
No (n=32)	HADS-A	11 (8)	11 (6)	0.444
	HADS-D	9 (6)	8 (5)	0.973
Yes (n=8)	HADS-A	10 (8)	10 (6)	0.235
	HADS-D	7 (3)	6 (7)	0.128

[1] Presented as median (interquartile range)

[2] Wilcoxon Signed Rank Test

[3] LLDAS, low disease activity state defined according to Franklyn *et al*.[1]

[4] LDA, low disease activity defined according to Polachek *et al*.[2]

Supplementary Table S3. Reclassification of the anxiety and depression level in association with longitudinal change in disease activity in SLE patients

	Group	No. patients	Change in SLEDAI-2K [1]	P value [2]
Anxiety level	Improved	9	-5 (6)	0.262
	Stable	18	-2 (2)	
	Worsened	13	-2 (4)	
Depression level	Improved	9	0 (2)	0.269
	Stable	24	-2 (4)	
	Worsened	7	-3 (4)	

[1] Median (interquartile range); [2] Kruskal-Wallis test (comparison across the three groups)

Supplementary Table S4. Anxiety and depression in association with sociodemographic and clinical characteristics of SLE patients

	HADS-A <11 [1]	HADS-A ≥11 [2]	P value [3]	HADS-D <8 [1]	HADS-D ≥8 [2]	P value [3]
Age (years) [4]	48.9 (10.0)	51.9 (10.6)	0.363	49.9 (9.8)	51.2 (11.2)	0.684
SLE duration (years) [4]	8.7 (6.8)	11.6 (7.1)	0.200	8.9 (6.5)	11.9 (7.5)	0.186
SLEDAI-2K [5]	6.0 (4.0)	6.0 (4.0)	0.745	6.0 (4.0)	6.0 (4.0)	0.649
No. comorbidities [5]	1.0 (2.0)	2.0 (3.0)	0.290	1.0 (3.0)	1.0 (2.0)	0.668
Organ damage (SDI) [5]	0.0 (1.0)	1.0 (1.0)	0.498	0.0 (1.0)	0.0 (1.0)	0.957
Gender (female) [6]	94.7%	100.0%	0.475	95.5%	100.0%	1.000
Education level [6]						
Primary	11.2%	19.0%	0.548	9.5%	22.4%	0.440
Secondary	44.4%	52.4%		47.6%	50.0%	
Higher/tertiary	44.4%	28.6%		42.9%	27.8%	
Working (yes) [6]	73.7%	33.3%	0.011	68.2%	33.3%	0.028

[1] n=19; [2] n=21; [3] Independent samples t-test (for normally distributed data) or Mann-Whitney test (for ordinal-type or non-normally distributed data); [4] Data are presented as mean (standard deviation); [5] Data are presented as median (interquartile range); [6] Data are presented as frequencies (percentages)

1. Franklyn, K.; Lau, C.S.; Navarra, S.V.; Louthrenoo, W.; Lateef, A.; Hamijoyo, L.; Wahono, C.S.; Chen, S.L.; Jin, O.; Morton, S.; et al. Definition and initial validation of a Lupus Low Disease Activity State (LLDAS). *Ann Rheum Dis* **2016**, *75*, 1615-1621, doi:10.1136/annrheumdis-2015-207726.
2. Polachek, A.; Gladman, D.D.; Su, J.; Urowitz, M.B. Defining Low Disease Activity in Systemic Lupus Erythematosus. *Arthritis Care Res (Hoboken)* **2017**, *69*, 997-1003, doi:10.1002/acr.23109.
